# A Short Half-Life α_IIb_β_3_ Antagonist ANTP266 Reduces Thrombus Formation

**DOI:** 10.3390/ijms19082306

**Published:** 2018-08-06

**Authors:** Tong-Dan Liu, Shen-Hong Ren, Xue Ding, Zhou-Ling Xie, Yi Kong

**Affiliations:** 1 School of Life Science & Technology, China Pharmaceutical University, 24 Tong Jia Street, Nanjing 210009, China; 15211030546@stu.cpu.edu.cn (T.-D.L.); 1621030478@stu.cpu.edu.cn (S.-H.R.); 14211030564@stu.cpu.edu.cn (X.D.); 2 School of Pharmacy, China Pharmaceutical University, 24 Tong Jia Street, Nanjing 210009, China; zhoulingxie@hfut.edu.cn

**Keywords:** platelets, bleeding risk, antithrombosis, α_IIb_β_3_, rapid elimination

## Abstract

Integrin α_IIb_β_3_ plays a pivotal role in platelet aggregation. Three α_IIb_β_3_ antagonists have been approved by the Food and Drug Administration (FDA) for the treatment of cardiovascular diseases. Unfortunately, all of these three drugs can cause the side effect of severe bleeding. Therefore, developing a new α_IIb_β_3_ antagonist with low bleeding was needed. In the present study, we screened compounds by using a fibrinogen/integrin α_IIb_β_3_ enzyme-linked immunosorbent assay (ELISA), and a novel α_IIb_β_3_ antagonist ANTP266 was attained. The antithrombotic effects of ANTP266 were estimated by using two animal models, the bleeding risk was estimated by using a mice tail cutting assay, and the plasma half-life time was tested by LC-MS/MS. The results showed that ANTP266 potently decreased thrombosis formation, while not prolonging bleeding time at its effective dosage. The bleeding of ANTP266 reduced rapidly as time went on from 5 to 60 min, but tirofiban produced high bleeding continuously. The plasma half-life of ANTP266 in rats was 10.8 min. Taken together, ANTP266 is an effective antithrombotic agent with a low bleeding risk. The shorter bleeding time benefits from its short plasma half-life. ANTP266 could be a candidate for developing the α_IIb_β_3_ antagonist of rapid elimination for a patient undergoing percutaneous coronary intervention.

## 1. Introduction

Cardiovascular diseases (CVDs) are now recognized as the leading causes of death in the world. Thrombosis plays a key role in the development of this class of diseases [1,2]. Platelets are small cells circulating in blood vessels that can be activated when the vessel is injured and then aggregated to form a platelet plug to avoid bleeding. However pathological conditions can also trigger platelet activation, lead to platelet aggregation, and then incur cardiovascular disorders [3,4]. An amount of evidence has confirmed the pivotal role of platelet aggregation in pathological thrombus formation, which hints that inhibition of platelet aggregation is a good strategy in the treatment of CVDs [2].

Many studies have been launched on the molecular basis and signal transduction of platelet aggregation. Integrin α_IIb_β_3_, which is the most abundant adhesion receptor on the surface of platelets with numbers as high as 60,000–80,000 per platelet, has been revealed to have a pivotal role in the process of platelet aggregation. α_IIb_β_3_ is a transmembrane glycoprotein, composed of α_IIb_ and β_3_ subunit [5]. It has two states: resting and active state. In the resting state, the affinity of α_IIb_β_3_ for fibrinogen is low, while in its active state, the affinity is high [6]. When platelets are activated, α_IIb_β_3_ turns into the active conformation, and fibrinogen crosslinks platelets by α_IIb_β_3_ to form platelet plugs. Thus, α_IIb_β_3_ inhibitors can be used for antithrombosis therapy [7,8].

Since the 1970s, many α_IIb_β_3_ inhibitors have been identified, and three of them, including abciximab, eptifibatide, and tirofiban, have received Food and Drug Administration (FDA) approvals for the treatment of CVDs. But, all of them cause serious side effects, such as bleeding and thrombocytopenia, which have limited their usage in clinical settings [9,10]. In the present study, we reported a new α_IIb_β_3_ inhibitor ANTP266 with potent antithrombotic function, as well as low bleeding side effects.

## 2. Results

### 2.1. Fibrinogen/Integrin α_IIb_β_3_ ELISA Screening Assay

**Figure 1:**
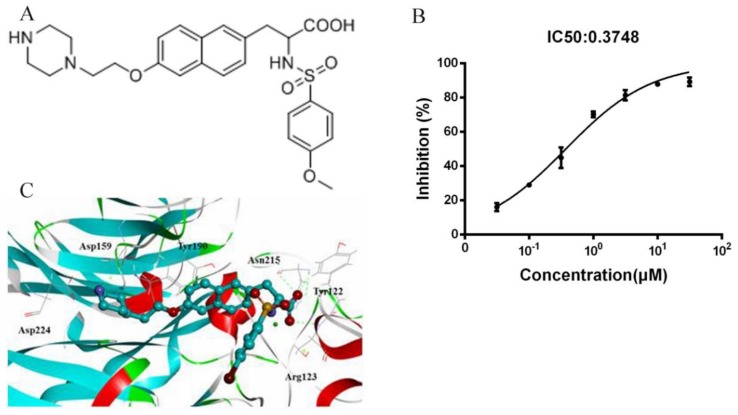
ANTP266 binding to integrin α_IIb_β_3_. (**A**) Chemical structure of naphthalene derivative ANTP266. (**B**) Effect of ANTP266 on the binding of fibrinogen to integrin α_IIb_β_3_. Purified human integrin α_IIb_β_3_ with or without ANTP266 was added to wells coated with fibrinogen for 2 h at 37 °C, followed by adding a mouse anti-human integrin β_3_ antibody. The binding of fibrinogen to α_IIb_β_3_ was determined using anti-mouse IgG-conjugated alkaline phosphatase and disodium 4-nitrophenyl substrate at 405 nm. All experiments were performed in triplicate. (**C**) Docking mode of ANTP266 to integrin α_IIb_β_3_ using CDOCKER in Discovery studio 3.0.

A series of naphthalene derivatives were analyzed using a fibrinogen/integrin α_IIb_β_3_ ELISA assay. Among these compounds, ANTP266 (Figure 1A) produced inhibition of α_IIb_β_3_ binding to fibrinogen in a dose-dependent manner with a half maximal inhibitory concentration (IC_50_) value of 0.37 μM (95%, 0.32–0.44 μM), as shown in Figure 1B.

### 2.2. Docking of ANTP266 to α_IIb_β_3_

To clarify whether ANTP266 can bind with α_IIb_β_3_, we performed a virtual molecular docking by using CDOCKER in Discovery studio 3.0. The result revealed that ANTP266 forms hydrogen bond interactions with Ser123 and Asn215, which is similar to those of tirofiban (Figure 1C and Figure S1). However, the basic group in ANTP266 piperazinyl forms hydrogen with Asp159, which is different from the interaction between piperidyl and Asp224 in the tirofiban complex.

### 2.3. ANTP266 Inhibited Human Platelet Aggregation In Vitro

**Figure 2:**
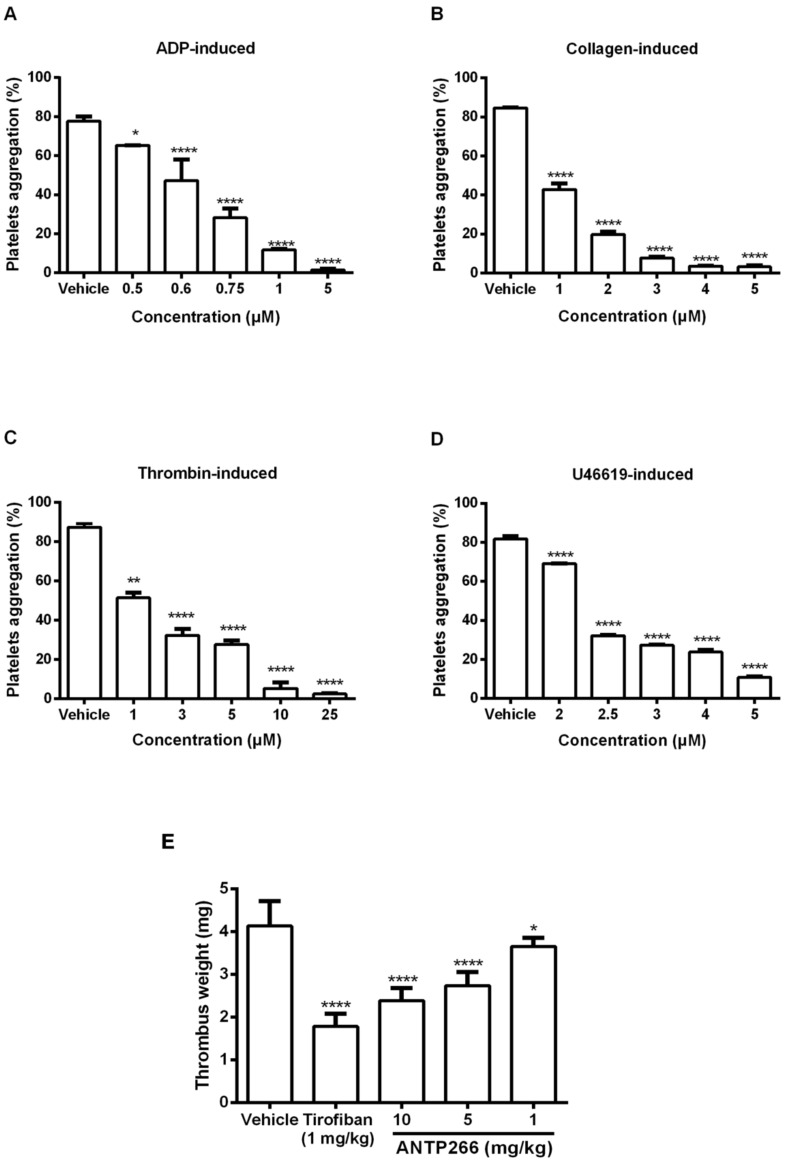
Effects of ANTP266 on human platelet activation in vitro and rat thrombosis in vivo. Human platelets were preincubated with different concentrations of ANTP266 or the vehicle for 5 min at 37 °C. Aggregation was initiated by the addition of 20 μM ADP (**A**), 1 μg/mL collagen (**B**), 0.25 U/mL thrombin (**C**) and 2 μM U46619 (**D**). Data are presented as mean ± SD (*n* = 3). * *p* < 0.05, ** *p* < 0.001 and **** *p* < 0.0001 compared with the vehicle, analyzed by one-way ANOVA, followed by the Dunnett multiple comparison test. (**E**) Effect of ANTP266 on arterio-venous shunt thrombosis in rats. ANTP266 (1, 5, 10 mg/kg), tirofiban (1 mg/kg), or the vehicle were administrated intravenously, and then, rats were anesthetized by intraperitoneal injection of chloral hydrate. The right carotid artery and left jugular vein of rat were linked by a tube with a silk thread in it. Data are the mean ± SD, * *p* < 0.05 and **** *p* < 0.0001 versus the vehicle, *n* = 6, analyzed by one-way ANOVA, followed by the Dunnett multiple comparison test.

Human platelet aggregation induced by various agonists were measured using a platelet aggregation analyzer. As shown in Figure 2A–D, ANTP266 inhibited human platelet aggregation induced by 20 μM ADP, 1 μg/mL collagen, 0.25 U/mL thrombin, and 2 μM U46619 with IC_50_ values of 0.67, 1.03, 1.62 and 2.60 μM, respectively.

### 2.4. ANTP266 Inhibited Thrombotic Formation In Vivo

To explore the antithrombotic activity of ANTP266 in vivo, ANTP266 was challenged in both an arterio-venous shunt thrombosis model in rats and an acute pulmonary embolism assay in mice. ANTP266 significantly inhibited thrombosis formation in a dose-dependent manner in the arterio-venous shunt thrombosis model. After administration of ANTP266 at 1, 5, and 10 mg/kg, thrombus weight reduced by 11.62 ± 5.02%, 33.82 ± 7.76%, and 42.29 ± 7.09% (mean ± SD, *n* = 6), respectively. At the dosage of 10 mg/kg, ANTP266 inhibited 42.29 ± 7.09% thrombosis, which was lower than tirofiban at 1 mg/kg with the inhibition rate of 56.82 ± 7.09% (Figure 2E).

In the acute pulmonary embolism model, thrombus stimulated by administration of ADP (300 mg/kg) occluded pulmonary vessels, incurring paralysis or death of the mice. Intravenous injection of the ANTP266 protected against the lethality of mice in a dose-dependent manner (Table 1). Administration of ANTP266 at a concentration of 10 mg/kg prevented paralysis and death with a protection rate of 90%, which was higher than that of tirofiban at 2.2 mg/kg (Table 1). These results indicated that ANTP266 effectively prevented thrombosis in vivo.

### 2.5. ANTP266 Inhibited Platelet Activation

**Figure 3:**
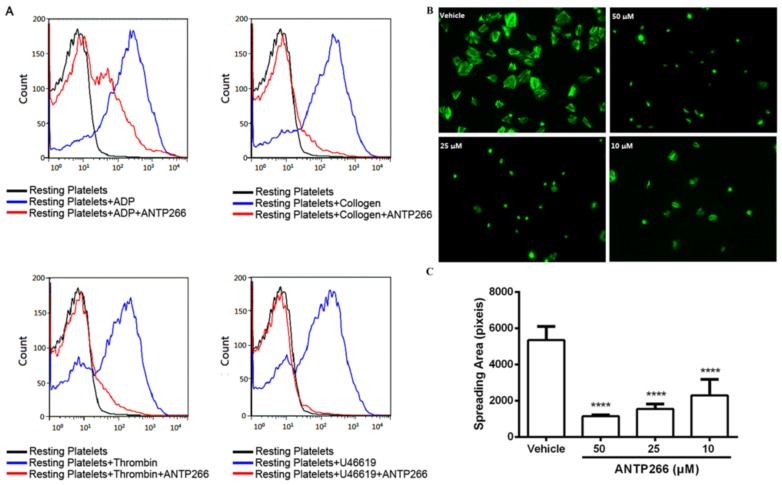
Effects of ANTP266 on the P-selectin expression and platelet spreading. (**A**) Effect of ANTP266 on the P-selectin expression. Platelet-rich plasma was preincubated with ANTP266 (50 μM) or the vehicle for 5 min at 37 °C. Activation was initiated by the addition of 20 μM ADP, 1 μg/mL collagen, 0.25 U/mL thrombin, or 2 μM U46619 for 5 min at 37 °C. P-selectin expression on platelet surface was detected by using flow cytometry. (**B**) Effect of ANTP266 on platelet spreading. Washed human platelets were allowed to adhere and spread on immobilized fibrinogen in a tissue culture incubator. After washing, the adherent platelets were fixed, permeabilized, stained, and captured under a fluorescence microscope. Spreading areas of adherent platelets were quantified using Metamorph software. Representative images fromat least three independent experiments with similar results. (**C**) Quantification of spreading areas (pixel number) of three random fields per experiment. Data are presented as mean ± SD (*n* = 3). **** *p* < 0.0001 compared with the vehicle.

P-selectin is a major marker protein of platelets activation. Various inducers were used to analyze the effect of ANTP266 on platelet activation, and the level of P-selectin expression was assessed by flow cytometry. As shown in Figure 3A, ANTP266 at a concentration of 50 μM potently inhibited P-selectin expression induced by 2 μM of U46619, 20 μM of ADP, 1 μg/mL of collagen, and 0.25 U/mL of thrombin with inhibition rates of 97.09%, 56.13%, 86.68%, and 82.38% respectively.

### 2.6. ANTP266 Inhibited Platelet Spreading

Outside-in signaling comes after the binding of a ligand with activated α_IIb_β_3_, which regulates platelet cytoskeletal reorganization and platelet spreading on immobilized fibrinogen [11]. We further investigated the effect of ANTP266 on the outside-in signaling, using platelets spreading assay. The result in Figure 3B,C showed that ANTP266 at concentrations of 50, 25, and 10 μM inhibited platelets spreading in a dose-dependent manner with inhibition rates of 78.75 ± 1.31%, 71.16 ± 5.29%, and 57.00 ± 16.39%, respectively, demonstrating that ANTP266 inhibited platelet activation by suppressing integrin α_IIb_β_3_-mediated outside-in signaling.

### 2.7. ANTP266 Exhibited a Low Bleeding Risk with a Short Plasm Half-Life

**Figure 4:**
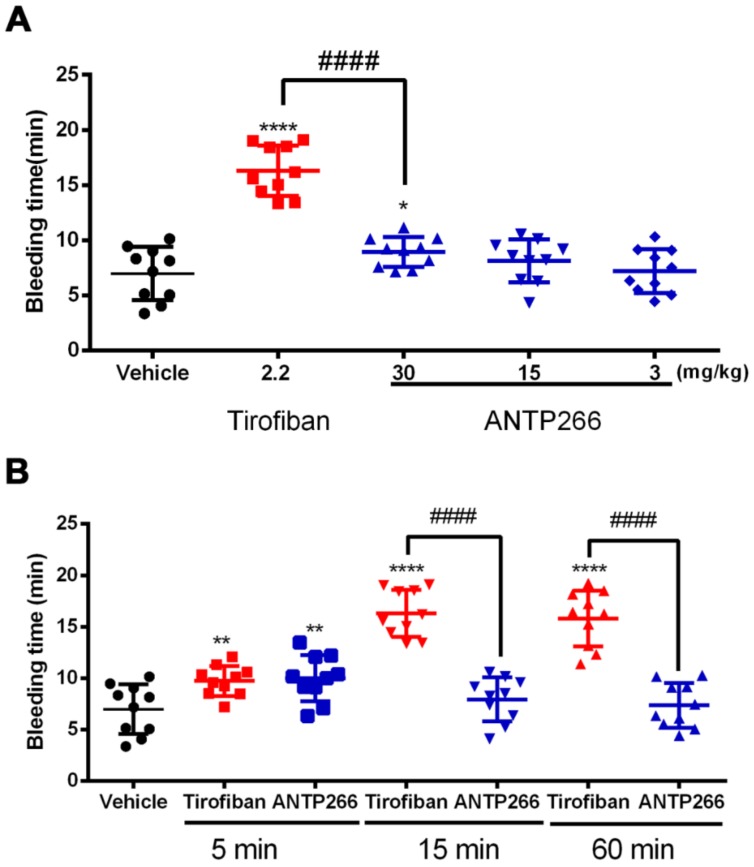
Effect of ANTP266 on bleeding time in mice. (**A**) Mice were administrated of ANTP266 (3, 15, 30 mg/kg), tirofiban (2.2 mg/kg) or the vehicle through caudal vein. Fifteen minutes later, a 3 mm-long tail tip was cut from the mice, the remaining tail was immersed immediately into saline at 37 °C. The accumulated bleeding time (including periods of re-bleeding) was recorded over a 20 min period. (**B**) Mice were administrated of 30 mg/kg ANTP266, or 2.2 mg/kg tirofiban. After 5, 15, or 60 min, the tip of mice tail was cut, and the bleeding time was tested as above. Data are presented as the mean ± SD (*n* = 10). * *p* < 0.05, ** *p* < 0.01 and **** *p* < 0.0001 Tirofiban or ANTP266 versus vehicle, ^####^ *p* < 0.0001 Tirofiban versus ANTP266, analyzed by one-way ANOVA, followed by the Dunnett multiple comparison test. Black: vehicle; Red: tirofiban; Blue: ANTP266.

To evaluate the bleeding incurred by ANTP266, we conducted a mice tail cutting assay with administration of ANTP266 at doses of 3, 15, and 30 mg/kg, which represented three times the dosages that were used in the anti-thrombotic mode. Tirofiban (2.2 mg/kg) was taken as a positive control. The results in Figure 4A showed that ANTP266 at 30 mg/kg slightly prolongated bleeding time (8.93 ± 1.36 min, mean ± SD, *n* = 10), which was shorter than that of tirofiban (16.30 ± 2.29 min, mean ± SD, *n* = 10) at 2.2 mg/kg. At doses of 15 and 3 mg/kg, which are three times the efficient dosages required to protect against paralysis or death in mice, ANTP266 did not significantly prolong the bleeding time (8.13 ± 1.94 min and 7.19 ± 1.99 min, respectively, mean ± SD, *n* = 10) compared with the vehicle group (6.99 ± 2.41 min), suggesting that ANTP266 confers a low bleeding risk.

In a further experiment, we investigated the time-dependent responsiveness of mice tail bleeding withANTP266 at 30 mg/kg and tirofiban at 2.2 mg/kg as a control. After administration for 5, 15, and 60 min, the tips of the mice tails were cut, and the accumulating bleeding times were observed within 20 min. As shown in Figure 4B, ANTP266 exhibited a slight bleeding risk after administration for 5 min (9.99 ± 2.23 min) but did not significantly prolong the bleeding time at 15 or 60 min (7.93 ± 2.14 min, 7.35 ± 2.17 min). Tirofiban at 5 min showed significant bleeding risk, and this tendency reached its peak value at 15 min and remained until 60 min (Figure 4B).

We investigated the potential reason for time-dependent low bleeding risk of ANTP266 by measuring its plasma half-life in rats using LC-MS/MS. The result showed that ANTP266 rapidly eliminated in plasma with a half-life time of 10.8 min (Table S1 and Figure S2), which was obviously shorter than that of tirofiban with 2 h [2].

## 3. Discussion

We found that ANTP266 restrained platelet aggregation stimulated by various agonists via interacting with α_IIb_β_3_. In vivo, ANTP266 showed potent antithrombotic effect with low bleeding, indicating that it is a promising compound for the development of antithrombotic drugs.

In normal blood circulation, platelets are in a “resting” state and activated when they come into contact with some adhesive proteins, such as collagen, as well as von Willebrand factor (VWF), and come into contact with platelet agonists, such as thrombin, ADP, and thromboxane A_2_. Integrin α_IIb_β_3_ on the surface of platelets is generally kept in a “resting” or low-affinity state and is transformed into an “activated” or high-affinity state after platelet activation [12,13]. Activated α_IIb_β_3_, by binding to its ligands (fibrinogen, VWF, and many matrix proteins containing RGD-like sequences), mediates platelet aggregation. ANTP266 showed strong inhibition of the binding of fibrinogen to α_IIb_β_3_, and the molecular docking results support that ANTP266 bound to α_IIb_β_3_. Activated α_IIb_β_3_ also mediates outside-in signaling transduction, leading to granule secretion, including P-selectin molecules (CD62P), platelet adhesion to collagen, platelet spreading, and clot retraction, which greatly amplifies platelet aggregation and thrombus size [14,15]. We identified that ANTP266 inhibited P-selectin expression by using flow cytometry and inhibited platelet spreading on fibrinogen by using fluorescence microscope. This evidence further supports that ANTP266 binds and inhibits integrin α_IIb_β_3_.

Inhibition of integrin α_IIb_β_3_ on the platelets lead to the decrease of platelet aggregation and reduction of thrombosis [10]. Three α_IIb_β_3_ inhibitors, including abciximab (ReoPro), tirofiban (Aggrastat), and eptifibatide (Integrilin), were approved by the FDA in 1994, 1998, and 1998, respectively, for the treatment of patients with Acute coronary syndrome (ACS), especially for those who are undergoing percutaneous coronary intervention (PCI) [9]. Unfortunately, all of them showed severe side effects in their clinical application, including intracranial and gastrointestinal bleeds and thrombocytopenia, which limited their extensive use in clinical settings. The use of α_IIb_β_3_ inhibitors has diminished in recent years compared with the broad use of them in the decade after their initial approval. But, given that α_IIb_β_3_ is a valid target, some researchers still concentrate on finding novel α_IIb_β_3_ antagonists that might not lead to bleeding risk and thrombocytopenia.

We reported here an α_IIb_β_3_ inhibitor ANTP266 with low bleeding risk and potent antithrombotic effects. At the same dose, ANTP266 showed a lower protection rate than tirofiban does in an acute pulmonary embolism model, but when the dose of ANTP266 increased (10 mg/kg), the protection rate was higher than that of tirofiban (2.2 mg/kg). Moreover, ANTP266 at 15 mg/kg did not show a significant bleeding risk, but tirofiban at 2.2 mg/kg showed obvious bleeding risk, indicating that ANTP266 has a better benefit/risk ratio.

In the in vivo bleeding test in mice, the bleeding time of ANTP266 decreased rapidly as time went on. A total of 15 min after administration, ANTP266 did not show bleeding risk while tirofiban continued to show high bleeding. It is expected that the drug administrated during the PCI could quickly be eliminated when the surgeries were finished so that it does not affect the normal function of platelets. To satisfy this anticipation, the short half-life of drug is desirable. In 2014, the FDA approved Cangrelor, a new P_2_Y_12_ inhibitor, as an adjunct to PCI owing to its fast onset and offset (plasma half-life of 3–6 min) and potent antithrombotic effect [16]. This approval implicated that the drug, which was administrated by intravenous infusion, with short half-life has some advantages over that of those with a longer half-life for a patient undergoing PCI. The half-life time of ANTP266 in rats was tested, and the results showed that the plasma half-life of ANTP266 is 10.8 min, which was obviously shorter than that of other α_IIb_β_3_ inhibitors: tirofiban, 2 h; abciximab, 2.5 h; and eptifibatide, 2.5 h [2]. Because of its short half-life, ANTP266 should be infused if it were to be used in clinical applications.

In conclusion, ANTP266 is a new αIIbβ3 antagonist that potently inhibited thrombosis but without increasing bleeding time. ANTP266 could be a candidate for the development of an agent for a patient who is undergoing PCI.

## 4. Materials and Methods

### 4.1. Reagents

ANTP266 2-((4-methoxyphenyl)sulfonamido)-3-(6-(2-(piperazin-1-yl)ethoxy)naphthalene-2-yl) propanoic acid was synthesized by Zhiyu Li (China Pharmaceutical University, China) and was dissolved in DMSO as a stock solution and stored at −20 °C. PE-conjugated anti-human CD62P, FITC-conjugated anti-human CD42a, REA Control (S)-PE, and REA Control (S)-FITC were from MiltenyiBiotec (Koln, Germany). The collagen was from Hyphen BioMed (Neuville sur Oise, France). Sepharose 2B beads, Aspirin, ADP, thrombin, U46619, human fibrinogen, apyrase, prostaglandin E1 (PGE1), [*N*-2-hydroxyethylpiperazine-*N*′-2-ethanesulfonic acid] (HEPES), biocytin, FITC-conjugated phalloidin, and anti-mouse IgG-conjugated alkaline phosphatase were purchased from Sigma Chemical Co. (St. Louis, MO, USA).

### 4.2. Animals

C57BL/6 wild-type mice and Sprague-Dawley rats were obtained from Nanjing Qinglongshan Animal Center. All experiments were carried out in accordance with the guidelines and the regulations of the Ethical Committee of the China Pharmaceutical University (No. S151010, 8 September 2015).

### 4.3. Interaction Assays between ANTP266 and Platelet α_IIb_β_3_ by ELISA

The analysis of interaction assays between ANTP266 and α_IIb_β_3_ was similar to the previous study with some modifications [4,17]. The 96-well microplate coated with fibrinogen was blocked with 1% albumin bovine serum (BSA) in TACTS for 1 h at 37 °C and then washed with TACTS. Human α_IIb_β_3_ solution and ANTP266 or 1% BSA in TACTS were added and incubated for 2 h at 37 °C. After washing, mouse anti-human β_3_ antibody was incubated for 1 h at 37 °C. Excess β_3_ antibodies were washed and anti-mouse IgG-conjugated alkaline phosphatase was added with incubation for 1 h at 37 °C. Disodium 4-nitrophenyl phosphate substrate was added after washing. After 30 min of substrate conversion, the reaction was stopped by adding 3 M of NaOH. The OD values of the samples were estimated at 405 nm.

### 4.4. Docking Studies

The binding modes of ligands (ANTP266 and tirofiban) with α_IIb_β_3_ were analyzed by CDOCKER in Discovery Studio 3.0 (Neotrident Technology Ltd., Beijing, China). Random ligand conformations were generated through molecular dynamics, and an exhaustive translational and rotational search of each conformation was carried out to generate low-energy orientations of the ligand within the active site of the rigid receptor. The final ligand conformations of ANTP266 were sorted by CHARMm energy (interaction energy plus ligand strain), and the crystal structure of α_IIb_β_3_ (PDB entry code: 2VDM) was extracted from the protein database. The ligands were docked in all possible stereoisomeric forms in an active site located in a sphere with 12 Å radius for α_IIb_β_3_, which was generated with the Create Sphere function around the subsequently removed crystal structure ligand. Docking for the ligands was performed, and the conformer with the lowest CHARMm energy was chosen for interpreting the docking results.

### 4.5. Platelet Aggregation in Aggregometer

Platelet aggregation was measured as previously described [18]. Briefly, human platelet-rich plasma (PRP) was obtained by centrifugation of anticoagulated human blood at 168× *g* for 5 min. Washed human platelets were prepared by centrifugation of the PRP at 953× *g* for 5 min in the presence of 1 U/mL of Apyrase and 0.1 μg/mL of PGE1 [19,20]. Platelet pellets were suspended with modified Tyrode’s solution and adjusted to 3 × 10^8^ platelets/mL. Platelets were incubated with ANTP266 or the vehicle for 5 min at 37 °C. The maximum platelet aggregation rate was determined by a lasting measurement of light transmittance for 5 min.

### 4.6. P-Selectin Expression

The expression of P-selectin on the platelet surface was determined by using flow cytometry according to the method described previously [21]. Briefly, human PRP was pre-incubated with ANTP266 or the vehicle for 10 min at 37 °C, and the platelets were separately stimulated with various agonists at 37 °C for 10 min. Then, the treated platelets were incubated with anti-CD62P-PE and anti-CD42a-FITC at 4 °C for 15 min at room temperature in the dark. After washing and resuspending in modified Tyrode’s buffer, the platelets were fixed with 1% paraformaldehyde at 4 °C. A flow cytometric study was performed on a BD FACSCalibur flow cytometer (Becton Dickinson, San Jose, CA, USA). For each sample, 20,000 platelets were analyzed.

### 4.7. Platelet Spreading on Fibrinogen-Coated Surface

Platelet spreading was measured as previously described [22]. Washed human platelets were preincubated with ANTP266 or the vehicle at 37 °C for 5 min. Treated platelets were allowed to spread on the fibrinogen-coated surfaces at 37 °C for 1 h. After three washes with phosphate buffer saline (PBS), the platelets were fixed, permeabilized, and stained with FITC-conjugated phalloidin. Immobilized platelets were visualized using an upright fluorescent microscope AXIO ScopeA1 (ZEISS Group, Jena, Germany) with a 100 × 3/1.50 oil objective lens, X-cite 120Q light source (EXFO, Mississauga, CA, USA), and a digital camera. The surface area of the platelets was analyzed using National Institutes of Health Image J software (National Institutes of Health, Bethesda, MD, USA) with pixel numbers as the unit of size.

### 4.8. In Vivo Arterio-Venous Shunt Thrombosis

A rat arterio-venous shunt thrombosis model was used to determine the antithrombotic activity of ANTP266 in vivo as described previously with a minor modification [4,8]. Sprague-Dawley rats were randomized into five groups and injected 1 mg/kg of tirofiban, ANTP266 (1, 5, 10 mg/kg), and the vehicle by group. After administration for 15 min, the rats were anesthetized, and two polyethylene tubes were installed into the right carotid artery and the left jugular vein, respectively. The arterio-venous shunt tubes with a silk thread in it linked the right carotid artery and the left jugular vein of the rat. After sustaining retained extracorporeal circulation of blood for 20 min, the thread was taken out for weighing. The dry weight of thrombus was measured after drying for 30 min at 60 °C by subtracting the weight of the dry 10 cm thread.

### 4.9. Tail-Bleeding Assay

Bleeding time was assessed by a tail cutting method as described previously with some modifications [17,23]. Briefly, mice administrated with ANTP266 or tirofiban were anesthetized with 10% chloral hydrate. A portion of tail tip (~3 mm) was amputated using a razor blade, and the remaining tail was immediately immersed into warmed saline (37 °C). Bleeding time was calculated within 20 min.

### 4.10. ADP-Induced Acute Pulmonary Thrombosis in Mice

Acute pulmonary thrombosis incurred with ADP in mice was similar to as previously described [24]. Mice were randomly divided into six groups, with 10 mice per group. After pre-treatment with intravenous injection of ANTP266 (at doses of 1, 2.2, 5, and 10 mg/kg), tirofiban (2.2 mg/kg) or with the vehicle for 5 min, the mice were challenged with ADP (300 mg/kg) through tail vein injection. The mice were observed to record the death and paralysis respectively within 15 min.

### 4.11. Detection of ANTP266 in Plasma

The rats were administrated with 10 mg/kg of ANTP266 by intravenous injection. A total of 200 microliters of blood was drawn after administration at different time points and collected into a heparinized tube. The blood samples were centrifuged at 4000× *g* for 15 min at 4 °C, and then, the plasma was precipitated by methanol. The supernatant was tested using LC-MS/MS as follows. The chromatography was performed on an HPLC system (comprised of 2 model LC-20AD pumps, a SIL-20AD autosampler, a CBM-20A system controller, and a CTO-20A oven, Shimadzu, Japan) coupled witha TSQ Quantum Ultra mass spectrometric detector with electrospray ionization (ESI) interface (Thermo Scientific, Waltham, MA, USA). The method was carried out on a VP-ODS-C_18_ column (2.0 mm × 150 mm, SHIMADUZ, Kyoto, Japan). All the processing, acquiring, and analysis of data were controlled using Xcalibur software (Thermo Scientific, Waltham, MA, USA). The HPLC separation was achieved on an isocratic mobile phase consisting of water (20%) and methanol (80%) at a flow rate of 1.0 mL/min with the column temperature maintained at 30 °C. The selected reaction monitoring (SRM) conditions were defined as follows: spray voltage, 5.0 kV; and capillary temperature, 350 °C. The SRM mode of *m*/*z* 541.01 → 113.10 [M + H]^+^ for ANTP266 and *m*/*z* 285.1 → 193.2 [M + H]^+^ for IS (diazepam) at positive ionization mode were used as quantitative analysis [25].

### 4.12. Statistical Analysis

The data were expressed as means ± SD and statistical analyses were undertaken using Prism 6.0 (GraphPad, San Diego, CA, USA). The differences between the groups were determined by one-way analysis of variance assay (ANOVA) followed by Dunnett’s test, and they were considered to be significant if *p* values were <0.05.

## Figures and Tables

**Table 1:** ANTP266 inhibited ADP-induced acute pulmonary thrombosis in mice.

	Dose (mg/kg)	Number of Deaths/Total	Number of Paralysis/Total	Protection Rate (%)
Vehicle	-	10/10	0/10	0
Tirofiban	2.2	1/10	1/10	80
ANTP266	2.2	2/10	2/10	60
	10	1/10	0/10	90
	5	2/10	1/10	70
	1	1/10	4/10	50

## Supplementary Materials

Supplementary materials can be found at http://www.mdpi.com/1422-0067/19/8/2306/s1.

## Abbreviations

FDA	Food and Drug Administration
CVDs	Cardiovascular diseases
ELIS	Enzyme linked immunosorbent assay
PGE1	Prostaglandin E1
HEPES	[*N*-2-hydroxyethylpiperazine-*N*′-2-ethanesulfonic acid]
PRP	Platelet-rich plasma
IgG	Immunoglobulin G
BSA	Albumin bovine serum
PE	P-phycoerythrin
FITC	Fluorescein isothiocyanate
HPLC	High performance liquid chromatography
ESI	Electrospray ionization
SRM	Selected reaction monitoring
VWF	Von Willebrand factor
PCI	Percutaneous coronary intervention
