# Supplementary material for: A Short Half-Life αIIbβ3 Antagonist ANTP266 Reduces Thrombus Formation
Source: Int J Mol Sci. 2018 Aug 6;19(8):2306. doi: 10.3390/ijms19082306 (PMC6121621; doi:10.3390/ijms19082306)
Supplement: Supplementary file 1 [file ijms-19-02306-s001.pdf]

A short half-life $\alpha_{IIb}\beta_3$ antagonist ANTP266 reduces thrombus formation without increased bleeding risk

Tong-dan Liu [1], Shen-hong Ren [1,], Xue Ding [1], Zhou-ling Xie [2], Yi Kong [1,*]

[1] School of Life Science & Technology, China Pharmaceutical University, 24 Tong Jia Street, Nanjing 210009, China
[2] School of Pharmacy, China Pharmaceutical University, 24 Tong Jia Street, Nanjing 210009, China
* Correspondence: yikong@cpu.edu.cn

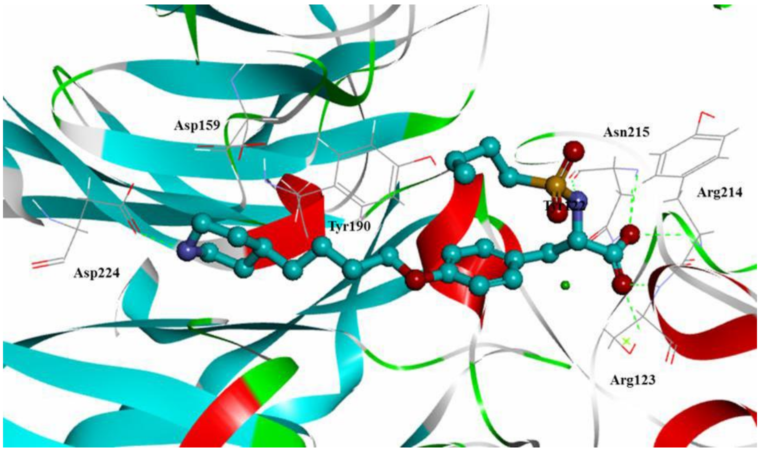

Figure S1. Docking mode of tirofiban binding to integrin $\alpha_{IIb}\beta_3$ using CDOCKER in Discovery studio 3.0.

Table S1. Plasma pharmacokinetic parameters of ANTP266 in rats after intravenous injection at 10 mg/kg (n = 5).

**AUC_{0-24} (μg/L•h)**	**$AUC_{0-\infty}$ (μg/L•h)**	**$T_{1/2}$ (h)**	**T_{max} (h)**	**CL_z (L/h/kg)**	**V_z (L/kg)**	**C_{max} (μg/L)**
14441.54 ±5659.55	14448.70 ±5656.71	0.18 ± 0.07	0.03 ± 0.00	776.22 ± 269.36	33.42 ± 26.10	99676.79 ±26265.66
